# Spotlight on early-career researchers: an interview with Benjamin Bitler

**DOI:** 10.1038/s42003-019-0380-z

**Published:** 2019-04-23

**Keywords:** Careers, Lab life, Ovarian cancer

## Abstract

Dr. Benjamin Bitler began his independent career at University of Colorado in January 2017. In this short Q&A he tells us about his motivation and passion for research, advice he would give to his younger self, and the attributes of cancer cells that he finds most perplexing.

**Figure:**
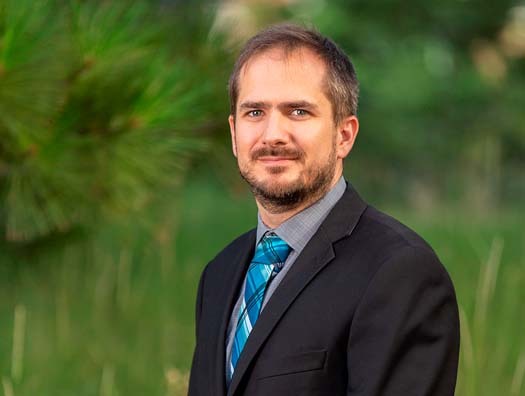
Image credit: Benjamin Bitler

Please tell us about your research interests.

The Bitler lab focuses on addressing critical clinical and basic science questions of ovarian cancer biology. We currently have several projects attempting to define mechanisms that promote ovarian cancer progression and targeted therapy resistance. For these projects, we are interested in identifying the influence of the epigenetic, immune, and metabolic microenvironments on ovarian cancer. For example, our recent work established Chromobox 2 (CBX2) as a novel epigenetic effector contributing to transcriptional reprogramming of metastatic ovarian cancer cells. The lab works closely with the University of Colorado Gynecologic Oncologists, which allows us to maintain crucial clinical perspective in our projects. Our goal is to establish pre-clinical data that will facilitate the translation of key findings.

What has your journey been to this point?

My journey, while unique, is likely more common than I appreciate. From early childhood, my goal was to “help others,” and as I entered high school, I had decided to work towards becoming a medical doctor. However, as in science, life often takes you down unintended paths. During my junior year in high school, my mom (age 46) was diagnosed with stage IV breast cancer; and while she fought valiantly, she ultimately lost her battle with the disease. During this time, I witnessed first-hand the devastation of chemotherapy, and I began to recognize how much the medical profession still did not understand about breast cancer. This close, personal, and tragic experience with cancer continued to direct my future because “helping others” took on a deeper meaning. If I could spare, or lessen this inexplicable suffering for someone else, that is what I wanted to do.

Therefore, upon entering college, my focus shifted to clinical oncology. During my undergraduate career at the University of Arizona, I was afforded the opportunity to volunteer in a research lab where I worked on the speciation of cactophilic fruit flies. Astonishingly, working with fruit flies is where I fell in love with research and lab culture. I finished my undergraduate degree and maintained a focus on oncology, which led me to apply to the University of Arizona Cancer Biology program. Remarkably, I received my acceptance letter to the Cancer Biology Program on the anniversary of my mom’s passing which appeared to be a sign that I was on the right track.

In the fall of 2005, I started my graduate education in cancer biology. I was extremely fortunate to have joined Dr. Joyce Schroeder’s lab, whose research interests, to develop new therapies and to better understand breast cancer, aligned with my own. Like most of my graduate school experiences, mine was marked with both failed experiments and likewise incredible successes. Ultimately, I completed my Ph.D. in the fall of 2010, thanks in part to the fantastic support and mentorship. Throughout my graduate career, I fell more in love with research and was encouraged to continue my doctoral training at another research institute where I could benefit from seeing research from another perspective. To this end, I accepted a post-doctoral position at the Fox Chase Cancer Center in Philadelphia, PA.

At Fox Chase Cancer Center, I joined Dr. Rugang Zhang’s lab and started my post-doctoral career. While Dr. Zhang’s lab focused on ovarian cancer, a disease that is ten years behind breast cancer with respect to scientific understanding and typical treatment options, there is some etiological overlap. There, I pursued several research projects examining signaling pathways and epigenetic regulation in ovarian cancer. I became passionate about epigenetic regulation and the potential of therapeutically targeting epigenetic processes. Just like during graduate school, my post-doctoral training included lessons on dealing with rejection and perhaps undeserved criticism from reviewers. Success in terms of accomplishments is often sharpened by those who both challenge and encourage you and during my post-doctoral fellowship, Dr. Aird (a fellow post-doc), as well as Dr. Zhang filled these roles admirably. My research in Dr. Zhang’s lab propelled me to a National Institute of Health Pathway to Independence award. Then, in 2016, I began my faculty search and was fortunate to be offered a position at the University of Colorado in the Division of Reproductive Sciences.

In January 2017, I became a Principal Investigator of my own lab in the Division of Reproductive Sciences. Now, I am currently in the midst of establishing my research program by continuing to examine ovarian cancer biology, where I hope to make significant strides in adding to the body of knowledge surrounding this disease. Throughout my journey, I have been blessed with incredible mentors and friends, which remains true in my current position. I realized a key to my research career success was to surround myself with people that were more talented and “smarter” than myself. I am grateful for the opportunity to affect change in the field of ovarian cancer, but it is also essential for me to provide opportunities to mentor and teach young researchers.

What are your predictions for your field in the near future?

My prediction is that in the near future, researchers will define a more holistic understanding of ovarian cancer tumorigenesis and progression. As researchers, we have tendencies to focus on one or two aspects of cancer biology, but the future lies in a more comprehensive understanding of the disease. For example, we are currently working to determine the role of metabolism and immune surveillance in ovarian cancer establishment. Defining the interplay between these major biological processes should uncover therapeutic vulnerabilities. Furthermore, I predict that increased implementation of tumor single-cell sequencing will improve our understanding of the tumor microenvironment complexity and will promote discovery of novel therapies that account for multiple aspects of tumor survival.

Can you speak of any challenges that you have overcome?

A significant challenge that I have overcome in my career is recognizing and admitting when I am not *the* expert on every topic. At every level of my education, I have had a problem or experiment that goes beyond my own proficiency. In these instances, one is often faced with two options: first, take the time to painstakingly exhaust all available information, thereby becoming an expert on the given subject or secondly, walk down the hall and consult an established expert. During my career, both scenarios have played out, and both have proven to be valuable experiences; but I caution anyone regarding “re-inventing the wheel” without talking to a collaborator first. For the research community to make significant discoveries it requires collaboration!

What advice would you give to your younger self?

I would tell my younger self two things: “TALK to people” and “do not let indecision and fear of failure paralyze you.”

A typical stereotype of researchers is that they are socially inept and have difficulty communicating. A part of this stereotype was true for me, and I feel like it held me back in some instances. I have endeavored to overcome this in several ways. For example, when searching for a reagent, I will intentionally visit every lab in the division, talk to the lab personnel, and discuss research with them. These experiences have opened doors and established unexpected collaborations.

Research is, unfortunately, more often defined by the failed experiments than the successful ones. As a young researcher, my indecision and fear of failure hindered my progress. Now, I implore the researchers in my lab to proceed with an experiment even when failure is likely. I would argue that seeing the result of a new technique for the first time, is one of the most gratifying experiences in research. “Failure” in research is inevitable, but the fear of such, should not prevent us from making progress.

What attribute of cancer cells still baffles you?

The attribute of cancer that still baffles and also amazes me is the sheer complexity and heterogeneity of the disease. I often wonder if some cancers respond to therapy as an independent organism. Within the tumor microenvironment, what is the level of coordination between the cancer cells and different cell types (fibroblast/immune cells) that potentially attenuates or exacerbates therapeutic responses? There certainly is a need to develop more complex models to further appreciate and account for cancer’s complexity.

*This interview was conducted by Associate Editor Yomayra Guzmán*

